# A High‐Pressure Praseodymium Fluoride Borate Linking Multiple Structural Features of Apatite‐Type Compounds

**DOI:** 10.1002/chem.201805092

**Published:** 2019-01-09

**Authors:** Matthias Glätzle, Almut Pitscheider, Oliver Oeckler, Klaus Wurst, Hubert Huppertz

**Affiliations:** ^1^ Institut für Allgemeine Anorganische und Theoretische Chemie Universität Innsbruck Innrain 80–82 6020 Innsbruck Austria; ^2^ Institut für Mineralogie, Kristallographie und Materialwissenschaft Universität Leipzig Scharnhorststraße 20 04275 Leipzig Germany

**Keywords:** apatite, borates, high-pressure chemistry, lanthanides, solid-state structures

## Abstract

Pr_5_(BO_4_)_3−*x*_(BO_3_)_*x*_(F,OH)_2.67_O_0.28_ (*x*≈1.6), a boron‐containing fluoride‐oxoapatite‐like compound, was obtained by the application of high‐pressure/high‐temperature synthesis. It exhibits a superstructure of the apatite type with a tripled *c* lattice parameter (space group *P*6_3_/*m*) and shows complex anion disorder along the 6_3_ screw axis and occupation of distorted octahedra, as well as almost trigonal planar sites, by oxygen and fluorine atoms. Furthermore, a distinct BO_4_/(BO_3_+F) group disorder is found; 46 % of the sites being occupied by BO_4_ groups and 54 % by BO_3_ groups, with a fluoride ion located near the missing oxygen atom. The rare earth cations in the 4*f* sites exhibit a specific distorted tricapped trigonal prismatic coordination with a mean metaprism twist angle of 21.3°. The crystal structure of Pr_5_(BO_4_)_3−*x*_(BO_3_)_*x*_(F,OH)_2.67_O_0.28_ (*x*≈1.6) shows much “flexibility” resulting in split and off‐site positions of all other rare earth cations. The title compound therefore combines many structural features of apatite‐like compounds, for example biologically highly‐important carbonated apatites, shedding more light onto the complex structural chemistry of apatites.

## Introduction

Due to their enormous biological significance as main mineral components of human and animal hard tissues such as bones and teeth, apatite Ca_5_(PO_4_)_3_(F,Cl,OH) and compounds with similar structures have been objects of extensive studies. Calcium phosphate biomaterials have been especially investigated as potential implant materials, or as scaffolds for growth factors in bone regeneration processes.^1^ Recently, Barheine et al. have reported that disordered borate‐containing apatite is responsible for the adhesion properties of organic molecules like proteins, and shows enhanced biodegradability in contrast to crystalline hydroxyapatite.^2^ Besides their biomedical features, the catalytic and piezoelectric properties of numerous apatites are well known.^3^ Apatite‐type compounds containing rare earth elements show interesting optical behaviour, and are potentially applicable as fluorescent lamp phosphors and laser‐material hosts.^4^ Recently, rare earth containing apatite‐type oxide ion conductors like La_8_Y_2_Ge_6_O_27_ have come into focus as potential electrolytes for solid oxide fuel cells (SOFC).^5^

The apatite prototype Ca_5_(PO_4_)_3_F was first reported by Navay et al.^6^ in 1930 and was confirmed to adopt *P*6_3_/*m* symmetry.^7^ To date, a large variety of compounds with the apatite structure type (*M*1)_2_(*M*2)_3_(*A*O_4_)_3_ *X* are known.^8^ The metal cations *M* can be alkaline earth metals, rare earth elements, Cd or Cr, whereas for the anion *X* the substituents F, Cl, Br, OH, O, or S are found.^9^ The tetrahedrally coordinated atom *A* can either be a transition metal (V, Mn, Cr) or a group 14 or 15 element (Si, Ge, P, or As).^10^ Many apatitic compounds adopt structures in lower‐symmetric trimetric space groups like *P*6_3_, *P* $\bar{6}$ and *P* $\bar{3}$ , as well as monoclinic ones, for example, *P*2_1_/*m* and *P*2_1_.

The scope of the apatite family is broadened by the inclusion of less common *M*_5_(*A*O_5_)_3_ *X* and *M*_5_(*A*O_3_)_3_ *X* compounds, where the *A*O_4_ tetrahedra are replaced by groups with square pyramidal or trigonal planar coordinations. One perfect example is natural hydroxyapatite, building up two thirds of human bone material, where the phosphate groups PO_4_ are partially replaced by carbonate groups CO_3_. Finnemanite Pb_5_(AsO_3_)_4_Cl,^11^ a reduced form of mimetite Pb_5_(AsO_4_)_4_Cl,^12^ adopts an apatite structure with *P*6_3_/*m* symmetry and a complete replacement of the *A*O_4_ tetrahedra by AsO_3_ groups, with the arsenic atoms lying above the triangular oxygen plane. Up to now, only a few boron‐containing apatites have been described. In most of these structures, boron is present in trigonal planar BO_3_ groups that partially substitute the tetrahedral *A*O_4_ groups.^13^ In Ca_5_(BO_3_)_3_F, tetrahedral PO_4_ groups are fully substituted by planar BO_3_ groups, leading to a monoclinic structure related to fluoroapatite.^14^ Ito et al. reported about the incorporation of boron as BO_3_ groups in oxoapatite.^15^ As far as we know, La_10_(Si_3.96_B_1.98_O_4_)_6_O_2_ is the only compound in whose structure the position *A* is partially occupied by fourfold‐coordinated boron atoms.^16^

Furthermore, some apatite superstructures have been reported in literature, mainly based on a doubling of one lattice parameter. A good overview of the crystal chemistry of apatites including a list containing apatite superstructures is given in ref. 8. Iodo‐oxoapatite was reported by Henning et al.^17^ as the first modulated apatite exhibiting superstructure ordering along [0 0 1], based on partial ordering of oxygen and iodine ions resulting in a tripling of the *c* lattice parameter in space group *P*6_3_/*m*. The final structure description, however, still displays disorder as related to the stacking sequence of anions within the iodine‐oxygen columns, with the anions being split over two positions each.

Pr_5_(BO_4_)_3−*x*_(BO_3_)_*x*_(F,OH)_2.67_O_0.28_ (*x*≈1.6) represents a high‐pressure rare earth fluoride‐oxoapatite‐like compound exhibiting a superstructure featuring a unit cell tripled along [0 0 1], in combination with anion disorder along the 6_3_ screw axis and BO_4_/ (BO_3_+F) group disorder. This compound therefore combines the two topics described above in a way that has not been reported so far, and could be a good basis for further understanding of the crystal chemistry in natural hydroxyapatites.

## Experimental Section

**Synthesis**: Pr_5_(BO_4_)_3−*x*_(BO_3_)_*x*_(F,OH)_2.67_O_0.28_ (*x*≈1.6) was obtained from a 1:1:2 mixture of Pr_6_O_11_, PrF_3_, and B_2_O_3_ by applying high‐pressure/high‐temperature conditions of 11.5 GPa and 1573 K, utilizing a Walker‐type multianvil apparatus. The starting mixture was ground under argon atmosphere and filled into boron nitride crucibles, which were then positioned inside MgO octahedra and compressed by eight tungsten carbide cubes. Pr_5_(BO_4_)_3−*x*_(BO_3_)_*x*_(F,OH)_2.67_O_0.28_ (*x*≈1.6) was obtained in form of greenish crystals that are stable in air at ambient conditions. Further experimental details on the synthesis are provided in the Supporting Information.

**Powder X‐ray diffraction (PXRD)**: A PXRD pattern of a flat polycrystalline sample of Pr_5_(BO_4_)_3−*x*_(BO_3_)_*x*_(F,OH)_2.67_O_0.28_ (*x*≈1.6) was collected in transmission geometry using a Stoe Stadi P powder diffractometer (Stoe & Cie GmbH, Darmstadt, Germany) with Mo${}_{K\alpha {}_{1}}$ radiation (Ge(1 1 1) monochromator, *λ*=70.930 pm) operated at 50 kV and 40 mA. The powder diffraction pattern of Pr_5_(BO_4_)_3−*x*_(BO_3_)_*x*_(F,OH)_2.67_O_0.28_ (*x*≈1.6) was recorded in the 2*θ* range 2.0–45.0° with a step‐size of 0.010°. Lattice parameter refinement was carried out using the *INDEX & REFINE* tool of the Stoe WinX^POW^ software suite.^18^ The experimental PXRD pattern in comparison with the theoretical powder pattern based on single‐crystal diffraction data of Pr_5_(BO_4_)_3−*x*_(BO_3_)_*x*_(F,OH)_2.67_O_0.28_ (*x*≈1.6) is shown in Figure S1; refined lattice parameters from powder diffraction data are given in the Supporting Information.

**Single‐crystal X‐ray diffraction (SCXRD)**: Small single crystals of Pr_5_(BO_4_)_3−*x*_(BO_3_)_*x*_(F,OH)_2.67_O_0.28_ (*x*≈1.6) were isolated by mechanical fragmentation, and selected under an optical polarization microscope. In line with the known issue with crystal quality of mixed apatites, only one out of dozens of crystals proved to be suitable for detailed single‐crystal structure determination. SCXRD data were collected at 173 K with a Bruker D8 Quest diffractometer (Photon 100 detector) equipped with a microfocus source generator (Incoatec GmbH, Geesthacht, Germany) combined with multi‐layer optics (monochromatized Mo_Kα_ radiation, *λ*=71.073 pm). Semiempirical absorption correction based on equivalent reflections was applied with SADABS‐2014/5.^19^ Systematic absences and Laue symmetry indicated the hexagonal space groups *P*6_3_/*m* or *P*6_3_. The structure was solved with SHELXS19b (version 2013/1). Structure refinement (full‐matrix least‐squares against *F* ^2^) with SHELXL^20^ (version 2014/7) using Stoe X‐Step32^21^ (Revision 1.05b) was successful in *P*6_3_/*m*; lower symmetries including *P* $\bar{6}$ did not result in less disorder or lower residuals. Due to large and highly anisotropic displacement parameters of the boron atoms B1A and B2, as well as the oxygen atoms O4 and O5, these positions were better refined as split positions B1/B1A, B2/B2A, O4/F4, and O5/F5. The ratio of occupation for these split positions was determined by free refinement of the occupancy factors, followed by small manual adaption to yield equal thermal displacement parameters for the boron positions B1:B1A and B2:B2A. The corresponding anion sites O4, O5, F4, and F5 were coupled with the boron occupancies to represent the chemically reasonable (BO_3_+F)/ BO_4_ polyhedra. At this point, there was still significant electron density in the proximity of the cations Pr1, Pr2, and Pr4. Assuming that the strong anion disorder impacts the positions of the rare earth cations, Pr1 was offset by 21(1) pm from the position on the mirror plane, and Pr2 was also refined as a split position (*d=*22(1) pm). Pr4 was also refined as a split position beneath its original position on the $\bar{6}$ axis. These modifications led to a stable refinement and drastic improvement of the *R* values. In first approximation, the required F^−^/O^2−^ mixing ration was balanced by the disordered positions. In second approximation, the atomic positions on the 6_3_ axis with high occupancy (F1, F2, and F3) were refined isotropically as fluorine atoms; the remaining positions (O11, O21, O23, and O33) were refined isotropically as oxygen atoms. A comparison of the thermal displacement parameters showed higher values for the fluorine and lower values for the oxygen atoms. Due to the fact that we could not exclude partial occupation by oxygen atoms on the positions of F4 and F5 within the (BO_3_+F) groups, refinement of all seven possible positions on the 6_3_ axis with statistical occupation by F/O would not allow for an exact analysis of distribution. To maintain overall charge neutrality, final refinement cycles were performed using fixed occupancy factors based on the results of their free refinement. The positional parameters of all atoms except those located either on split positions or on the 6_3_ screw axis were refined with anisotropic displacement parameters. Final difference Fourier synthesis did not reveal any significant residual peaks. Due to the fact that oxygen and fluorine cannot be distinguished unequivocally by means of SCXRD data, the differentiation between O and F on the 6_3_ screw axis, and also within the (BO_3_+F) groups, is a reasonable model based on interatomic distances (see Table S4 in Supporting Information). Occupation of the F4 and F5 positions by oxygen atoms can almost certainly be excluded, due to the fact that the system would therefore relax forming the BO_4_ group. While not indicated by the single‐crystal structure data or infrared (IR) spectroscopy, the presence of OH groups or water molecules in the title compound cannot be excluded. Due to the preparation of the high‐pressure assembly at ambient conditions under air, a partial hydrolysis of the starting materials could occur. Furthermore, the presence of H_2_O and OH groups in oxoapatites and halogen‐oxoapatites is quite common.^22^ Therefore, we assume the composition of the title compound to be best‐represented with the formula Pr_5_(BO_4_)_3−*x*_(BO_3_)_*x*_(F,OH)_2.67_O_0.28_ (*x*≈1.6).

Relevant details of the data collection and evaluation are listed in Table S1. Positional parameters, anisotropic and equivalent isotropic displacement parameters, interatomic distances, and interatomic angles (within BO_3_/BO_4_ groups only) are provided in Tables S2‐S5. CCDC 1872320 contains the supplementary crystallographic data for Pr_5_(BO_4_)_3−*x*_(BO_3_)_*x*_(F,OH)_2.67_O_0.28_ (*x*≈1.6). These data are provided free of charge by The Cambridge Crystallographic Data Centre / FIZ Karlsruhe deposition service.

## Results and Discussion

**Figure 1:**
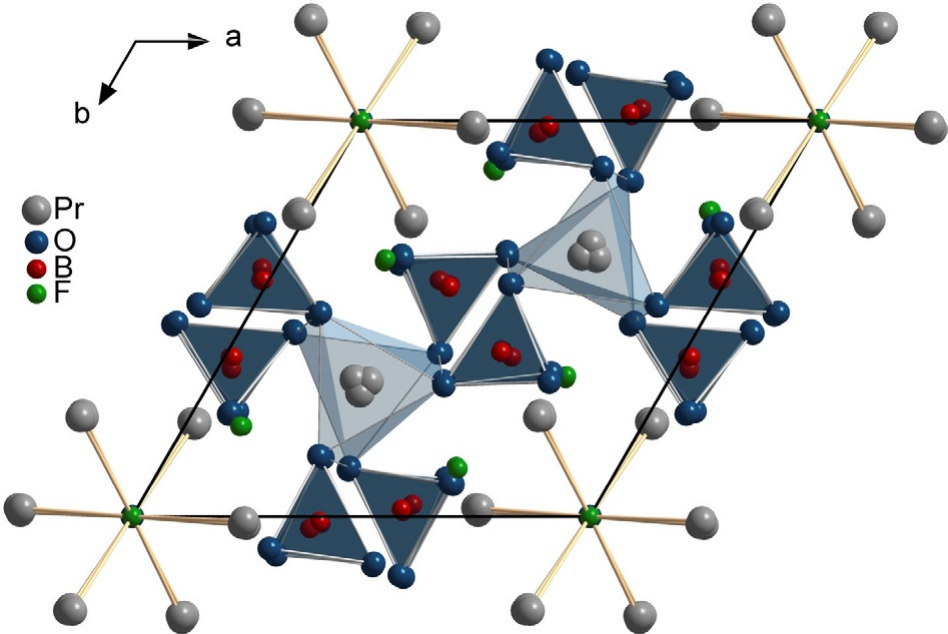
Ball‐and‐stick model representing the crystal structure of Pr_5_(BO_4_)_3−*x*_(BO_3_)_*x*_(F,OH)_2.67_O_0.28_ (*x*≈1.6) viewed along [0 0 1]. BO_4_ polyhedra are shaded in dark blue, PrO_6_ metaprisms in light blue.

Crystal structure refinement shows that Pr_5_(BO_4_)_3−*x*_(BO_3_)_*x*_(F,OH)_2.67_O_0.28_ (*x*≈1.6) crystallizes in the hexagonal centrosymmetric space group *P*6_3_/*m* with the lattice parameters *a=*918.4(2) pm and *c=*2163.1(4) pm. A classical representation of the apatitic crystal structure of Pr_5_(BO_4_)_3−*x*_(BO_3_)_*x*_(F,OH)_2.67_O_0.28_ (*x*≈1.6) is given in Figure 1, highlighting the BO_3_/BO_4_ groups. The rare earth cations surrounding the 6_3_ screw axis form trigonal planar, tetrahedral, and octahedral sites for the anions in the channels along [0 0 1], as well as the metaprisms formed by the rare earth cations Pr3 and Pr5.

**Figure 2:**
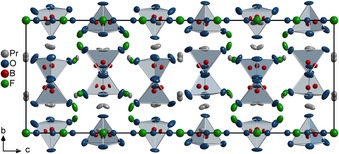
Representation of the apatite superstructure of Pr_5_(BO_4_)_3−*x*_(BO_3_)_*x*_(F,OH)_2.67_O_0.28_ (*x*≈1.6), displacement ellipsoids at 90 % probability level, viewed along [1 0 0], displaying a tripling of the *c* lattice parameter. Complex disorder of the anions in the channels along [0 0 1] as well as disorder of BO_4_/(BO_3_+F) groups are shown.

The superstructure involves tripling of the *c* lattice parameter with respect to the basic apatite structure type. A representation of the crystal structure with displacement ellipsoids at 90 % probability level (Figure 2) illustrates the nature of the superstructure within the unit cell, and depicts the rather strong anisotropic displacement of the rare earth cations as well as the oxygen atoms forming the BO_3_ and BO_4_ groups.

**Figure 3:**
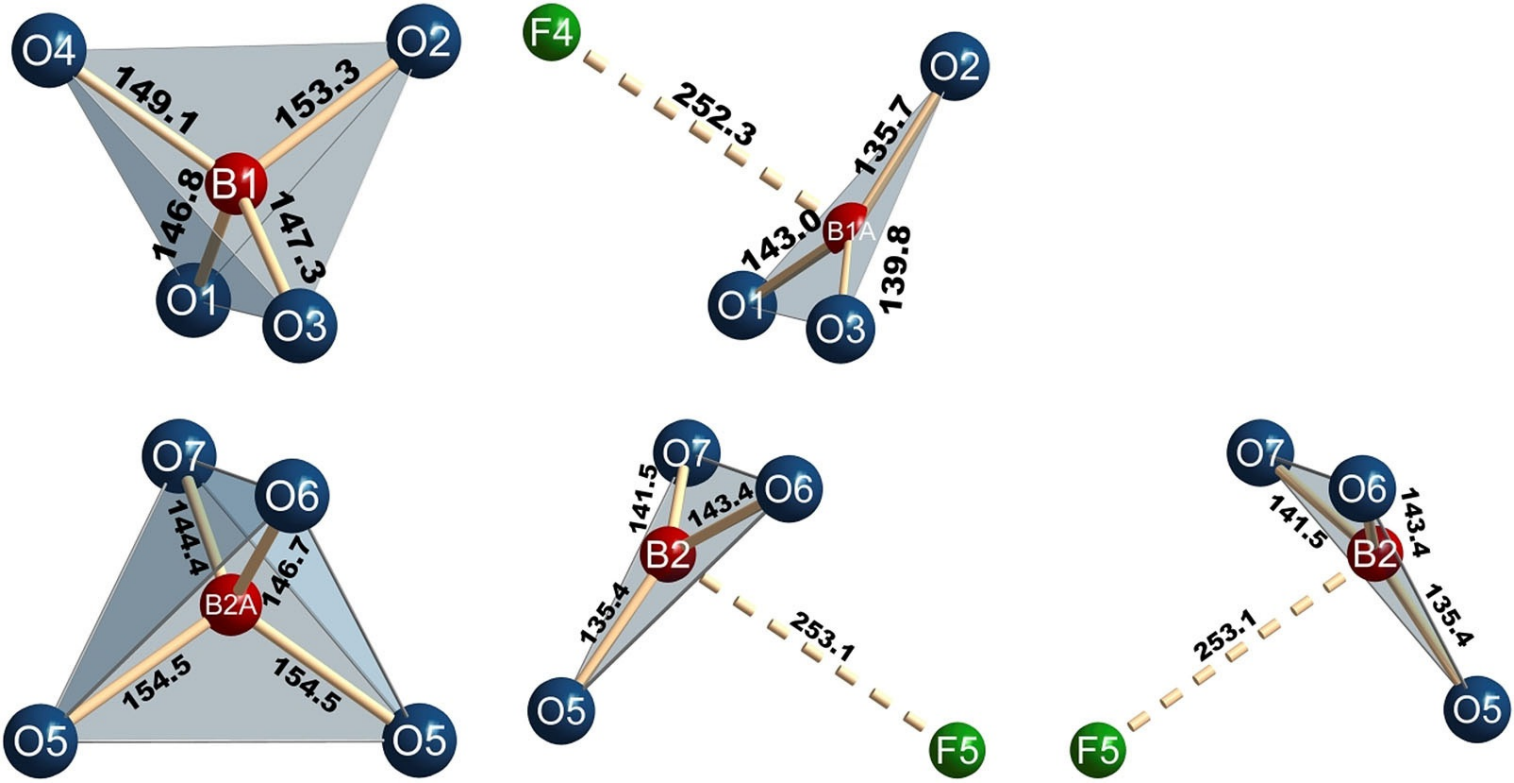
BO_4_/(BO_3_+F) group disorder in the crystal structure of Pr_5_(BO_4_)_3−*x*_(BO_3_)_*x*_(F,OH)_2.67_O_0.28_ (*x*≈1.6) including interatomic distances (in pm).

It is obvious that the superstructure arises from partial ordering of oxygen and fluorine atoms along [0 0 1], as well as the partial ordering of the BO_4_/(BO_3_+F) group disorder (Figure 3).

In fact, only the (B2)O_3_ groups are found on two faces of the tetrahedron, whereas the (B1A)O_3_ group is only located on one side of the tetrahedron. This is depicted in Figure 3, including all relevant interatomic distances. In all cases, only the adjacent planes of BO_3_ triangles oblique to [0 0 1] are occupied. Carbonate hydroxyapatites are known to show a similar *A*O_4_/*A*O_3_ group disorder, where trigonal planar CO_3_ groups partially substitute tetrahedral PO_4_ groups, forming one oxygen vacancy per substituting group. However, for these carbonate apatites, including human and animal tooth enamels, no concurring models are reported regarding the locations of vacancies on the oxygen sites of the tetrahedra. Depending on the positions of the vacancies (seen as reduced occupancy factors in the positional parameters of the crystal structure solution), a location of the faces of the tetrahedra, where the CO_3_^2−^ groups are partially located, is possible. For the calcium‐deficient carbonate hydroxyapatite Ca_3.40_[Ca_5.90_(NH_4_)_0.10_][(PO_4_)_4.95_(CO_3_)_1.05_(H_2_O)_0.30_][(OH)_1.65_(H_2_O)_0.45_], Ivanova et al. reported the carbonate ions to occupy those two adjacent faces of a PO_4_ tetrahedron, which are parallel to the *c*‐axis.^23^ However, polarized IR spectroscopy shows the planes of the CO_3_ triangles to be oblique to [0 0 1] at an angle of 37±4°.^24^ The latter is consistent with the positions of the BO_3_ groups in the crystal structure of Pr_5_(BO_4_)_3−*x*_(BO_3_)_*x*_(F,OH)_2.67_O_0.28_ (*x*≈1.6), with part of these groups additionally found on two planes and partly on only one of the four tetrahedral planes.

The oxygen atoms O3 in carbonate hydroxyapatite (equaling O1, O3, respectively O6, O7 in Pr_5_(BO_4_)_3−*x*_(BO_3_)_*x*_(F,OH)_2.67_O_0.28_ (*x*≈1.6)) are split in two positions (at a distance of 37 pm), depending on coordination by phosphorus as PO_4_^3−^ or by carbon as CO_3_^2−^.^23^ While this is not the case in Pr_5_(BO_4_)_3−*x*_(BO_3_)_*x*_(F,OH)_2.67_O_0.28_ (*x*≈1.6), we herein find sites near the positions of O4 and O5, which are partly occupied by fluorine atoms (F4 and F5). F4 is shifted 49(1) pm apart from O4, and F5 about 48(1) pm apart from O5, both in the direction off the center of the BO_4_ tetrahedra. The resulting positions and interatomic distances lead to the conclusion that the positions of F4 and F5 are occupied wherever the tetrahedral BO_4_ group is replaced by a BO_3_ group, as is shown in Figure 3. To the best of our knowledge, this has not been reported in an apatitic structure so far.

However, a similar structural situation can be found in solid‐solution fluoride borates like, for example, Eu_3_(BO_3_)_2+*x*_F_3−3*x*_,^25^ Ba_3.12_Sr_3.88_(BO_3_)_3.65_F_3.05_,^26^ and Ba_7_(BO_3_)_4−*x*_F_2+3*x*_ (*x=*0.49)26b, 27 with [(BO_3_)F]^4−^↔[F_4_]^4−^ anionic isomorphism. In these compounds, planar BO_3_ groups directly neighbored to a fluoride anion in trigonal pyramidal geometry, together with (4F)^4−^ groups, are statistically distributed over the crystal structure. The BO_3_ groups thereby can appear on each face except at the basal plane of the trigonal pyramid. In the crystal structure of Pr_5_(BO_4_)_3−*x*_(BO_3_)_*x*_(F,OH)_2.67_O_0.28_ (*x*≈1.6), the BO_3_ groups are only located at one or two planes of the tetrahedra (see above). While no (4F)^4−^ groups are present in the title compound, one could, in coherence with the (BO_3_)^3−^↔(3F)^3−^ anionic isomorphism, describe the disorder of BO_4_ and (BO_3_+F) groups in Pr_5_(BO_4_)_3−*x*_(BO_3_)_*x*_(F,OH)_2.67_O_0.28_ (*x*≈1.6) as [(BO_3_)F]^4−^↔[BO_4_]^5−^ anionic isomorphic substitution. Having a closer look at the distances between the boron atom of the BO_3_ groups to the fluoride anions, one can find values of 236(3) and 261(4) pm for Ba_3.12_Sr_3.88_(BO_3_)_3.65_F_3.05_ 26 and Ba_7_(BO_3_)_4−*x*_F_2+3*x*_ (*x=*0.49),26b, 27 respectively. This compares well to the corresponding B⋅⋅⋅F interatomic distances of 252.3(1) and 253.1(1) pm found in Pr_5_(BO_4_)_3−*x*_(BO_3_)_*x*_(F,OH)_2.67_O_0.28_ (*x*≈1.6).

Very recently, Li et al. reported the possible formation of tri‐coordinated planar triangle (PO_3_+O) groups in a mixed borate‐phosphate‐fluoride with the composition Ca_5−*y*_(BO_3_)_3−*x*_(PO_4_)_*x*_F (CBP_*x*_F):*y*Bi^3+^,^28^ when BO_3_ is gradually substituted by PO_4_. At the composition *x=*2.0–2.5 and *y=*0.5, 0.15, PO_4_ groups may partially be described as a planar PO_3_ group in combination with an isolated oxygen atom above the phosphorus atom. At *x*<1.2, the crystal structure of Ca_5−*y*_(BO_3_)_3−*x*_(PO_4_)_*x*_F (CBP_*x*_F):*y*Bi^3+^ is reported to correspond to Ca_5_(BO_3_)_3_F (space group *Cm*).^29^

The presence of BO_4_ as well as BO_3_ groups in the crystal structure of Pr_5_(BO_4_)_3−*x*_(BO_3_)_*x*_(F,OH)_2.67_O_0.28_ (*x*≈1.6) was confirmed by IR spectroscopy measurements of a bulk sample, as is presented in detail in the Supporting Information. The ratio of occupation with BO_4_ and (BO_3_+F) groups in Pr_5_(BO_4_)_3−*x*_(BO_3_)_*x*_(F,OH)_2.67_O_0.28_ (*x*≈1.6) was determined by refinement of the occupancy factors of the corresponding boron and oxygen atoms. This yielded 62 % BO_4_ and 38 % BO_3_ for B1 and B1A, and 16 % BO_4_ and 84 % BO_3_ for B2A and B2, respectively. Summed up, the ratio of BO_4_:BO_3_ in Pr_5_(BO_4_)_3−*x*_(BO_3_)_*x*_(F,OH)_2.67_O_0.28_ (*x*≈1.6) is 139:161, equaling 46 % BO_4_ and 54 % BO_3_ groups. The central atoms of the less frequent groups were termed with the suffix “A”.

The interatomic distances and angles in the BO_3_ and BO_4_ groups show values typical for slightly distorted tetrahedral and trigonal planar coordinations, respectively (Figure 3). The mean values of the interatomic distances, however, are slightly larger than expected, with 149.1 pm for (B1)O_4_, 150 pm for (B2A)O_4_ (literature value: 147.6 pm^30^), 139.5 pm for (B1A)O_3_, and 140.1 pm for (B2)O_3_ (literature value: 137.0 pm^31^). Partial substitution of oxygen in the BO_3_/BO_4_ groups by fluorine is therefore very unlikely, as this would result in even smaller mean interatomic distances than the literature values given above (mean literature value d(B−O/F) in tetrahedral BF_4_ groups=139 pm,^32^ in BO_2_F_2_=142.8 pm,^33^ and in BO_3_F=145.0 pm).33b, 34 Furthermore, calculations of the bond valence sums within the BO_4_ and BO_3_ groups have been performed according to the bond‐length/bond‐strength concept.^35^ These result in formal charges for the central boron atoms lower than +3 (B1=+2.90, B1A=+2.86, B2=+2.82, B2A=+2.83) and would decrease further by approximately 0.1 each for every oxygen atom being substituted by fluorine.

All praseodymium cations in the crystal structure of Pr_5_(BO_4_)_3−*x*_(BO_3_)_*x*_(F,OH)_2.67_O_0.28_ (*x*≈1.6) are in the trivalent state. While minor amounts of tetravalent praseodymium cations cannot be excluded by any method, there is no experimental evidence for significant amounts of tetravalent cations. The light‐green color of the product sample, as well as the relatively high mean interatomic distances (see Table S4) compared to literature values of the crystal radii clearly account for Pr^3+^ cations (sum of crystal radii^36^: *r*(Pr^3+^, C.N.=7)+*r*(O^2−^/F^−^, C.N.=4)=241 pm; *r*(Pr^3+^, C.N.=8)+*r*(O^2−^/F^−^, C.N.=4)=247.6 pm; *r*(Pr^4+^, C.N.=7)+*r*(O^2−^/F^−^, C.N.=4)=226 pm; *r*(Pr^4+^, C.N.=8)+*r*(O^2−^/F^−^, C.N.=4)=231 pm). For mixed valent rare earth oxides, for example Pr_9_O_16_ and Pr_10_O_18_, such comparison of mean interatomic distances to sums of crystal radii has been shown to be applicable for distinguishing between trivalent and tetravalent rare earth cations.^37^ Moreover, the title compound has been found to preferably form at the edge of the reaction vessel near the boron nitride crucible, which has a slightly reducing effect on the reaction sample.

**Figure 4:**
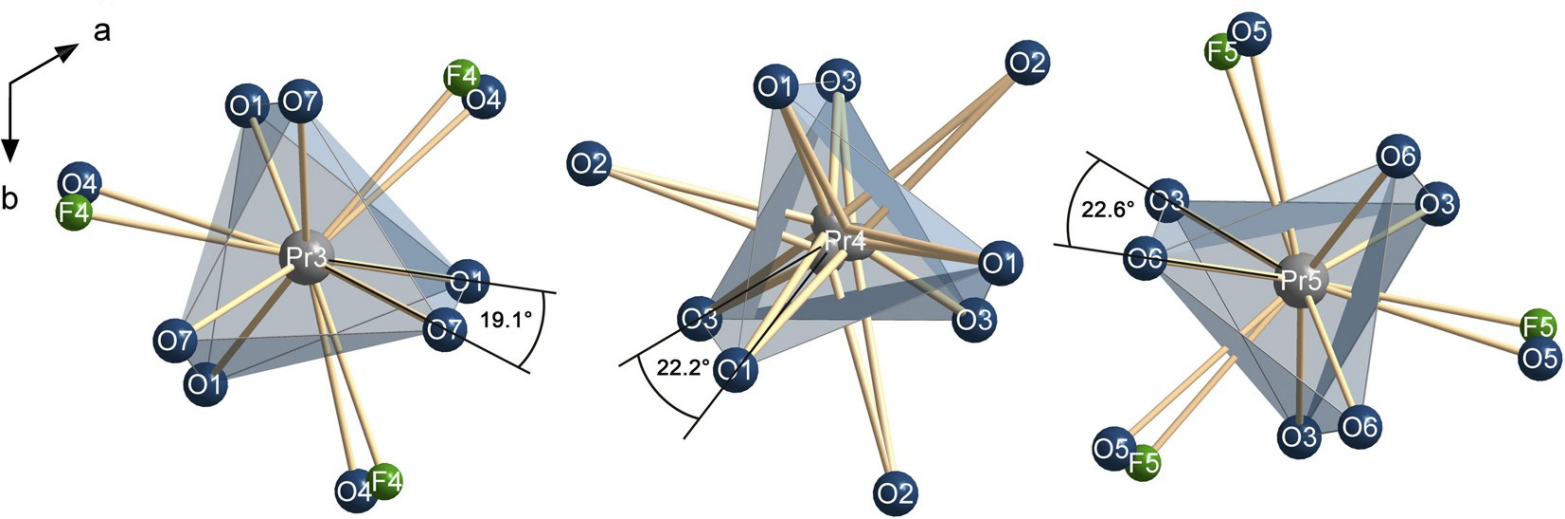
Tricapped trigonal metaprismatic coordination polyhedra of Pr3, Pr4, and Pr5 in Pr_5_(BO_4_)_3−*x*_(BO_3_)_*x*_(F,OH)_2.67_O_0.28_ (*x*≈1.6) shown along [0 0 1], highlighting the metaprism twist angles characteristic for these polyhedra in apatite‐type compounds.

The rare earth cations Pr3, Pr4, and Pr5 on the *M*1 positions show distorted tricapped trigonal prismatic coordination characteristic for the apatite structure, with the addition of the capping atoms of Pr3 and Pr5 being either oxygen (O4/O5) or fluorine (F4/F5), depending on the neighboring group being BO_4_ or (BO_3_+F) (Figure 4). The metaprism twist angles *ϕ* in Pr_5_(BO_4_)_3−*x*_(BO_3_)_*x*_(F,OH)_2.67_O_0.28_ (*x*≈1.6) are *ϕ*_Pr3_=19.1(1)°, *ϕ*_Pr4_=22.2(1)° and *ϕ*_Pr5_=22.6(1)°. *ϕ*_Pr3_ therefore equals the value found for chloroapatite of 19.1°, *ϕ*_Pr4_ and *ϕ*_Pr5_ are close to the twist angle of (*M*1)O_6_ polyhedra in fluoroapatite of 23.3°. This is in very good agreement with the observation of White et al. as that the metaprism twist angle *ϕ* of the (*M*1)O_6_ polyhedra in apatite‐type compounds varies inversely with average ionic radii and unit cell volume.^8^ Although no data are available for apatitic compounds with praseodymium as the cation *M*1, the nearly equal ionic radii of Pr^3+^ (C.N.=8: 127 pm; C.N.=9: 132 pm) and Ca^2+^ (C.N.=8: 126 pm; C.N.=9: 132 pm) enable good comparability of the mean metaprism angle obtained for Pr_5_(BO_4_)_3−*x*_(BO_3_)_*x*_(F,OH)_2.67_O_0.28_ (*x*≈1.6) (*V=*0.5266 nm^3^, corresponding to 1/3 of the supercell) of 21.3° with the values reported in literature for Ca_10_(PO_4_)_6_F_2_ (*V=*0.5226 nm^3^) of 23.3° and Ca_10_(PO_4_)_6_Cl_2_ (*V=*0.5376 nm^3^) of 19.1°.^8^

Indicated by large anisotropic displacement parameters, the rare earth cations Pr1 and Pr4 were positioned off their original positions on the mirror plane and the $\bar{6}$ axis, respectively, with equal partial occupancy each. Furthermore, the cation Pr2 was refined as split position (Pr2 and Pr2A) to comply with single‐crystal XRD data. Pr1 and Pr2 are each 7/8 coordinated, depending on the occupation of anions along [0 0 1]. This is similar to most common apatite‐type structures.

**Figure 5:**
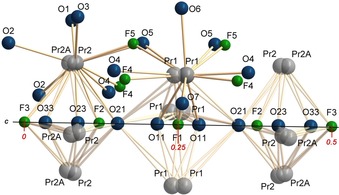
Disordered anion sites in the crystal structure of Pr_5_(BO_4_)_3−*x*_(BO_3_)_*x*_(F,OH)_2.67_O_0.28_ (*x*≈1.6) along the 6_3_ screw axis occupied by fluorine and to a minor extent with oxygen atoms (partial occupancy), indicating the possible coordination sites. Furthermore, the distorted pentagonal bipyramidal coordination spheres of the rare earth cations Pr2/Pr2A and Pr1 are depicted. Note that the distinction between F, O, and OH is tentative.

The anions in the channels along [0 0 1] are strongly disordered and their average positions in the crystal structure of Pr_5_(BO_4_)_3−*x*_(BO_3_)_*x*_(F,OH)_2.67_O_0.28_ (*x*≈1.6) can only be estimated due to the positional disorder, which is a well‐known phenomenon for apatite‐type structures. The possible sites of the oxygen and fluorine atoms along the 6_3_ screw axis, together with the resulting coordination spheres are shown in Figure 5. Whereas the positions of the fluorine atoms are almost fully occupied, the oxygen sites are only occupied to a minor fraction (see Table S2 in Supporting Information). Note that mainly every other octahedral and trigonal planar site is occupied (F3 and F1, respectively). In natural fluoroapatite, the fluoride ions occupy the sites 2*a* (*z*=1/4 , triangular interstices between Ca atoms) along [0 0 1] only, whereas the anions of apatites containing larger halides or hydroxides (hydroxyapatite) tend to occupy 2*b* (*z=*0, octahedral coordination by Ca atoms) or to statistically occupy 4*e* sites.

## Conclusion

By the application of high‐pressure/high‐temperature synthesis, Pr_5_(BO_4_)_3−*x*_(BO_3_)_*x*_(F,OH)_2.67_O_0.28_ (*x*≈1.6), a fluoride‐oxoborate with apatite‐like structure was obtained. The title compound exhibits complex disorder of anions along [0 0 1], as well as BO_4_/ (BO_3_+F) group disorder. Partial ordering results in an apatite superstructure with a tripled lattice parameter *c*. Pr_5_(BO_4_)_3−*x*_(BO_3_)_*x*_(F,OH)_2.67_O_0.28_ (*x*≈1.6) thereby combines structural features that have only been reported in different apatitic compounds, for example, the *A*O_4_/*A*O_3_ group disorder in carbonate hydroxyapatite, the main component of human and animal hard tissues, and the superstructure of iodo‐oxoapatite Ca_15_(PO_4_)_9_IO. The formation of BO_4_ tetrahedra instead of trigonal planar BO_3_ groups is preferred at high‐pressure conditions during the synthesis–known as the pressure‐coordination rule. Therefore, the synthesis of apatite structures with exclusively BO_4_ groups replacing the PO_4_ groups of the apatite prototypes will be an interesting objective for future research. Owing to their high potential as scaffolds of growth factors and carriers for controlled protein release for bone regeneration and for biodegradable bone implants, further research in high‐pressure synthesis of borate‐containing hydroxyapatites and their characterization will be of great interest.

## Conflict of interest

The authors declare no conflict of interest.

## Supporting information

As a service to our authors and readers, this journal provides supporting information supplied by the authors. Such materials are peer reviewed and may be re‐organized for online delivery, but are not copy‐edited or typeset. Technical support issues arising from supporting information (other than missing files) should be addressed to the authors.

Supplementary
