# Supplementary material for: A High‐Pressure Praseodymium Fluoride Borate Linking Multiple Structural Features of Apatite‐Type Compounds
Source: Chemistry. 2019 Jan 9;25(7):1767–72. doi: 10.1002/chem.201805092 (PMC6392139; doi:10.1002/chem.201805092)
Supplement: Supplementary file 1 — Supplementary [file CHEM-25-1767-s001.pdf]

CHEMISTRY
A European Journal

Supporting Information

A High-Pressure Praseodymium Fluoride Borate Linking Multiple Structural Features of Apatite-Type Compounds

Matthias Glätzle,[a] Almut Pitscheider,[a] Oliver Oeckler,[b] Klaus Wurst,[a] and Hubert Huppertz*[a]

chem_201805092_sm_miscellaneous_information.pdf

Synthesis

A polycrystalline sample of $Pr_5(BO_4)_{3-x}(BO_3)_x(F,OH)_{2.67}O_{0.28}$ ($x \approx 1.6$) was synthesized under high-pressure/high-temperature conditions of 11.5 GPa and 1573 K from a 1:1:2 mixture of Pr_6O_{11}, PrF_3, and B_2O_3 (all from Strem Chemicals, Newburyport, MA, USA, 99.9%). Powder X-ray diffraction of the starting materials showed no significant crystalline impurities. The starting mixture was finely ground under Ar inert gas atmosphere and filled into boron nitride crucibles (Henze BNP AG, Lauben, Germany), which were sealed with a boron nitride lid. However, the use of the same mixture stored at ambient conditions in air for eight days before the synthesis also led to the formation of the same products. The crucibles containing the reactants were positioned in the center of 18/11 assemblies, which were compressed by eight tungsten carbide cubes (Hawedia, ha-7%Co, Marklkofen, Germany). Details on the construction of the assembly are described in the literature.[1] Quasi-hydrostatic pressure was applied by a Walker-type multianvil module and a 1000 ton press (both Max Voggenreiter GmbH, Mainleus, Germany). Heating was supplied by a resistive graphite tube heating. The sample was compressed up to 11.5 GPa within 5 h, followed by heating it up to 1573 K in 10 min, keeping this temperature for another 10 min, and cooling down to 773 K within 20 min. After quenching to room temperature by switching off the heating, the decompression of the assembly required 14 h. The product was isolated by breaking apart the octahedral pressure medium (MgO, Ceramic Substrates & Components Ltd., Newport, Isle of Wight, UK) and careful separation from the surrounding graphite and boron nitride. $Pr_5(BO_4)_{3-x}(BO_3)_x(F,OH)_{2.67}O_{0.28}$ ($x \approx 1.6$) was obtained in form of green air- and water-resistant crystals.

Crystallographic Data

Table S1. Single-crystal X-ray diffraction data and structural parameters from refinement of $Pr_5(BO_4)_{3-x}(BO_3)_x(F,OH)_{2.67}O_{0.28}$ ($x \approx 1.6$) (standard deviations in parentheses).

Empirical formula	$Pr_5(BO_4)_{3-x}(BO_3)_x(F,OH)_{2.67}O_{0.28}$ ($x \approx 1.6$)
Formula weight	958.31
Crystal system	hexagonal
Space group	$P6_3/m$
Crystal color	green
**Single-crystal data**	
Single-crystal diffractometer	Bruker D8 Quest (Photon 100 detector)
Radiation; λ, pm	Mo-K_α; 71.073
a, pm	918.4(2)
c, pm	2163.1(4)
V, nm³	1.5799(5)
Formula units per cell (Z)	6
Calculated density, g cm^{-3}	6.04
Crystal size, mm³	0.12 × 0.09 × 0.05
Temperature, K	173(2)
Absorption coefficient, mm^{-1}	22.8
F(000), e	2516
θ range, °	2.6-37.5
Range in *hkl*	±15, ±15, ±37
Total no. of reflections	57055
Independent reflections / R_{int}	2837 / 0.0389
Reflections with I > $2\sigma(I)$ / R_σ	2595 / 0.0132
Data / ref. parameters	2837 / 145
Absorption correction	semiempirical
	(Bruker SADABS 2014/5)[2]
GoF on F^2	1.229
R_1 / wR_2 [$I > 2\sigma(I)$]	0.0265 / 0.0604
R_1 / wR_2 (all data)	0.0302 / 0.0615
Residual density, / 10^{-6} e pm^{-3}	1.41 / -2.49

Table S2. Atomic coordinates, occupancy factors, and isotropic or equivalent isotropic displacement parameters (U_{eq} in Å²) for $Pr_5(BO_4)_{3-x}(BO_3)_x(F,OH)_{2.67}O_{0.28}$ ($x \approx 1.6$) (space group $P6_3/m$) based on single-crystal X-ray diffraction data. U_{eq} is defined as one third of the trace of the orthogonalized U_{ij} tensor.

Atom	Wyckoff position	*x*	*y*	*z*	Occupancy factor	U_{eq} / U_{iso}
Pr1	12*i*	0.76191(4)	0.74011(3)	0.24048(2)	½	0.00700(9)
Pr2	12*i*	0.7497(4)	0.9887(4)	0.0826(2)	½	0.0076(3)
Pr2A	12*i*	0.7490(4)	0.9798(4)	0.0732(2)	½	0.0073(2)
Pr3	4*f*	⅔	⅓	0.16208(2)	⅓	0.00580(5)
Pr4	12*i*	0.6879(4)	0.347(2)	0.00051(2)	⅓	0.0080(3)
Pr5	4*f*	⅓	⅔	0.16618(2)	⅓	0.00562(5)
O1	12*i*	0.5868(3)	0.1182(3)	0.0790(2)	1	0.0094(4)
O2	12*i*	0.3399(3)	0.0812(3)	0.0273(2)	1	0.0136(4)
O3	12*i*	0.3311(3)	0.8466(3)	0.0819(2)	1	0.0107(4)
O6	6*h*	0.4124(4)	0.8756(4)	¼	½	0.0092(5)
O7	6*h*	0.6578(4)	0.1484(4)	¼	½	0.0116(6)
B1	12*i*	0.4029(6)	0.0302(6)	0.0844(2)	0.615[a]	0.0050(7)
B1A	12*i*	0.415(1)	0.013(1)	0.0613(4)	0.385 [a]	0.005(2)
O4	12*i*	0.3625(7)	0.0968(6)	0.1407(2)	0.615	0.0099(7)
F4	12*i*	0.3551(8)	0.1260(8)	0.1585(3)	0.385	0.011(1)
B2	12*i*	0.5793(9)	0.9820(9)	0.2270(3)	0.42 [a]	0.004(1)
B2A	6*h*	0.597(3)	0.970(3)	¼	0.08 [a]	0.005(4)
O5	12*i*	0.6532(9)	0.9165(8)	0.1910(3)	0.58	0.013(1)
F5	12*i*	0.6508(8)	0.8787(8)	0.1755(3)	0.42	0.011(1)
F1	2*a*	1	1	¼	0.142[b]	0.013(1)
F2	4*e*	1	1	0.1214(2)	0.2582 [b]	0.0129(8)
F3	2*b*	1	1	0	0.129 [b]	0.014(2)
O11	4*e*	1	1	0.216(2)	0.0247 [c]	0.008(8)
O21	4*e*	1	1	0.151(2)	0.0324 [c]	0.008(6)
O23	4*e*	1	1	0.087(2)	0.0427 [c]	0.008(5)
O33	4*e*	1	1	0.034(2)	0.0376 [c]	0.009(5)

[a] refined to nearly equal isotropic thermal displacement parameters
[b,c] refined to nearly equal isotropic thermal displacement parameters whilst taking into account overall charge neutrality

Table S3. Anisotropic equivalent displacement parameters U_{ij} (Å²) for $Pr_5(BO_4)_{3-x}(BO_3)_x(F,OH)_{2.67}O_{0.28}$ ($x \approx 1.6$) (space group $P6_3/m$) based on single-crystal X-ray diffraction data.

Atom	U_{11}	U_{22}	U_{33}	U_{23}	U_{13}	U_{12}
Pr1	0.0066(1)	0.00333(9)	0.0112(3)	-0.00248(8)	-0.00402(9)	0.00255(8)
Pr2	0.0060(2)	0.0033(2)	0.0128(7)	-0.0005(4)	-0.0007(4)	0.0019(2)
Pr2A	0.0031(2)	0.0063(5)	0.0112(6)	0.0031(4)	0.0002(4)	0.0013(3)
Pr3	0.00684(7)	0.00684(7)	0.00372(9)	0	0	0.00342(3)
Pr4	0.0104(9)	0.007(2)	0.0069(2)	0.0006(4)	-0.0004(2)	0.005(2)
Pr5	0.00705(7)	0.00705(7)	0.00275(9)	0	0	0.00352(3)
O1	0.0044(8)	0.0078(9)	0.016(1)	-0.0009(7)	0.0020(7)	0.0030(7)
O2	0.021(2)	0.0118(9)	0.0136(9)	-0.0057(8)	-0.0096(8)	0.0126(9)
O3	0.0107(9)	0.0036(8)	0.018(1)	0.0014(7)	-0.0019(7)	0.0035(7)
O6	0.002(1)	0.005(2)	0.020(2)	0	0	0.0013(9)
O7	0.006(2)	0.003(2)	0.024(2)	0	0	0.0012(9)
O4	0.012(2)	0.009(2)	0.008(2)	0	0.003(2)	0.004(2)
F4	0.010(2)	0.010(3)	0.007(3)	0.002(2)	-0.003(2)	0.002(2)
O5	0.020(2)	0.013(2)	0.010(2)	0.003(2)	0.008(2)	0.011(2)
F5	0.008(2)	0.013(3)	0.009(3)	0.003(2)	0	0.003(2)

Table S4. Interatomic distances (in pm) for $Pr_5(BO_4)_{3-x}(BO_3)_x(F,OH)_{2.67}O_{0.28}$ ($x \approx 1.6$) ($P6_3/m$, single-crystal data, standard deviations in parentheses). Due to the disorder of the crystal structure and partial occupancies (see Table S2), this list of interatomic distances seems to imply higher coordination numbers than locally adopted in the crystal structure.

Pr1	F4	199.8(6)	Pr2	F5	222.8(7)	Pr2A	O23	223.8(5)
	F1	230.24(4)		O23	225.1(4)		O2	229(3)
	O4	231.8(5)		O1	233.2(4)		O33	238(2)
	O11	235(2)		F2	240(4)		F5	239.5(7)
	F4	237.1(6)		O5	247.8(6)		O1	239.7(4)
	O6	238.5(3)		O2	247.9(4)		O2	244.6(4)
	F5	243.7(7)		O33	248(2)		F2	245.2(4)
	O11	248(2)		O2	249.2(3)		O3	258.1(4)
	O5	252.6(6)		F4	252(7)		O5	266.5(6)
	O7	263.7(3)		O4	254(6)		O4	270.5(6)
	F5	269.5(7)		O3	263.3(4)		F4	270.8(7)
	O5	272.6(6)		O21	270(2)		F3	272.5(3)
	**Ø**	**243.5**		F3	287.2(3)		O21	279(3)
				**Ø**	**249.3**		**Ø**	**252.1**
Pr3	3× O1	249.4(2)	Pr4	O1	230.5(3)	Pr5	3× O3	246.7(2)
	3× O7	252.4(2)		O3	233.4(4)		3× O6	247.0(2)
	3× F4	252.4(7)		O3	246(1)		3× F5	258.0(7)
	3× O4	258.2(5)		O1	248.9(9)		3× O5	272.8(7)
	**Ø**	**253.1**		O1	249.1(9)		**Ø**	**256.1**
				O3	253.4(7)			
				O2	268(2)			
				O2	273.(2)			
				O2	294.9(4)			
				**Ø**	**258.2**			
B1	O1	146.8(6)	B1A	O2	135.7(8)	B2	O5	135.4(9)
	O3	147.3(6)		O3	139.8(8)		O7	141.5(8)
	O4	149.1(7)		O1	143.0(8)		O6	143.4(8)
	O2	153.3(5)		**Ø**	**139.5**		**Ø**	**140.1**
	**Ø**	**149.1**						
B2A	O7	144(3)						
	O6	147(3)						
	2× O5	155(2)						
	**Ø**	**150**						

Table S4. (continued)

O1		B1A	143.0(8)	O11	3×	Pr1	235(2)	O2		B1A	135.7(8)
		B1	146.8(6)		3×	Pr1	248(2)			B1	153.3(5)
		Pr4	230.5(3)			**Ø**	**241.5**			Pr2A	229.0(3)
		Pr2	233.2(4)							Pr2A	244.6(4)
		Pr2A	239.7(4)							Pr2	247.9(4)
	2×	Pr4	249.1(9)							Pr2	249.2(3)
		Pr3	249.4(2)							Pr4	268(2)
		**Ø**	**217.6**							Pr4	273(2)
										Pr4	294.9(4)
										**Ø**	**232.8**
O21	3×	Pr2	270(2)	O23	3×	Pr2A	223.8(5)	O33	3×	Pr2A	238(2)
	3×	Pr2A	279(3)		3×	Pr2	225.1(4)		3×	Pr2	248(2)
		**Ø**	**274.5**			**Ø**	**224.5**			**Ø**	**243**
O3		B1A	139.8(8)	O4		B1	149.1(7)	O5		B2	135.4(9)
		B1	147.3(6)			Pr1	231.8(5)			B2A	155(2)
		Pr4	233.4(4)			Pr2	254.0(6)			Pr2	247.8(6)
		Pr4	246(1)			Pr3	258.2(5)			Pr1	252.6(6)
		Pr5	246.7(2)			Pr2A	270.5(6)			Pr2A	266.5(6)
		Pr4	253.4(7)			Pr1	270.6(5)			Pr1	272.6(6)
		Pr2A	258.1(4)			**Ø**	**239.0**			Pr5	272.8(7)
		Pr2	263.3(4)							**Ø**	**229.0**
		**Ø**	**223.5**								
O6	2×	B2	143.4(8)	O7	2×	B2	141.5(8)	F1	4×	Pr1	230.24(4)
		B2A	147(3)			B2A	144(3)		2×	Pr1	230.25(4)
	2×	Pr1	238.5(3)		2×	Pr3	252.4(2)			**Ø**	**230.2**
	2×	Pr5	247.0(2)		2×	Pr1	263.7(3)				
		**Ø**	**200.7**			**Ø**	**208.5**				
F2	3×	Pr2	240.0(4)	F3	6×	Pr2A	272.5(3)	F4		Pr1	199.8(6)
	3×	Pr2A	245.2(4)		6×	Pr2	287.2(3)			Pr1	237.1(6)
		**Ø**	**242.6**			**Ø**	**279.9**			B1A	252(1)
										Pr2	252.0(7)
										Pr3	252.4(7)
										Pr2A	270.8(7)
										**Ø**	**244.0**
F5		Pr2	222.8(7)								
		Pr2A	239.5(7)								
		Pr1	243.7(7)								
		B2	253(1)								
		Pr5	258.0(7)								
		Pr1	269.5(7)								
		**Ø**	**247.8**								

Table S5. Interatomic angles (in °) within the BO_4 and the BO_3 groups in the crystal structure of $Pr_5(BO_4)_{3-x}(BO_3)_x(F,OH)_{2.67}O_{0.28}$ ($x \approx 1.6$) ($P6_3/m$, single-crystal data, standard deviations in parentheses).

O1	B1	O3	111.2(4)	O2	B1A	O3	123.2(6)
O1	B1	O4	107.0(4)	O2	B1A	O1	118.6(6)
O3	B1	O4	116.0(4)	O3	B1A	O1	118.2(6)
O1	B1	O2	105.9(3)			**Ø**	**120.0**
O3	B1	O2	107.4(3)				
O4	B1	O2	108.9(4)				
		**Ø**	**109.4**				
O5	B2	O7	125.7(6)	O7	B2A	O6	110.3(18)
O5	B2	O6	120.0(6)	O7	B2A	O5	111.0(12)
O7	B2	O6	114.0(5)	O6	B2A	O5	106.5(12)
		**Ø**	**119.9**	O7	B2A	O5	111.0(12)
				O6	B2A	O5	106.5(12)
				O5	B2A	O5	111.3(18)
						**Ø**	**109.4**

Powder X-ray diffraction

The experimental powder X-ray diffraction pattern of the reaction product (Figure S1) is consistent with the theoretical powder pattern simulated from single-crystal X-ray diffraction data. However, the experimental powder pattern shows some additional reflections assignable to $Pr_{12}B_{11}O_{31}F_7$[3] (marked with asterisks) and the starting materials (marked with circles). Residual reflections belong to an unidentified side phase. By indexing the reflections of $Pr_5(BO_4)_{3-x}(BO_3)_x(F,OH)_{2.67}O_{0.28}$ ($x \approx 1.6$), we obtained the parameters a = 919.8(2), c = 2158.9(3) pm, and a volume of 1.5818(3) nm³.

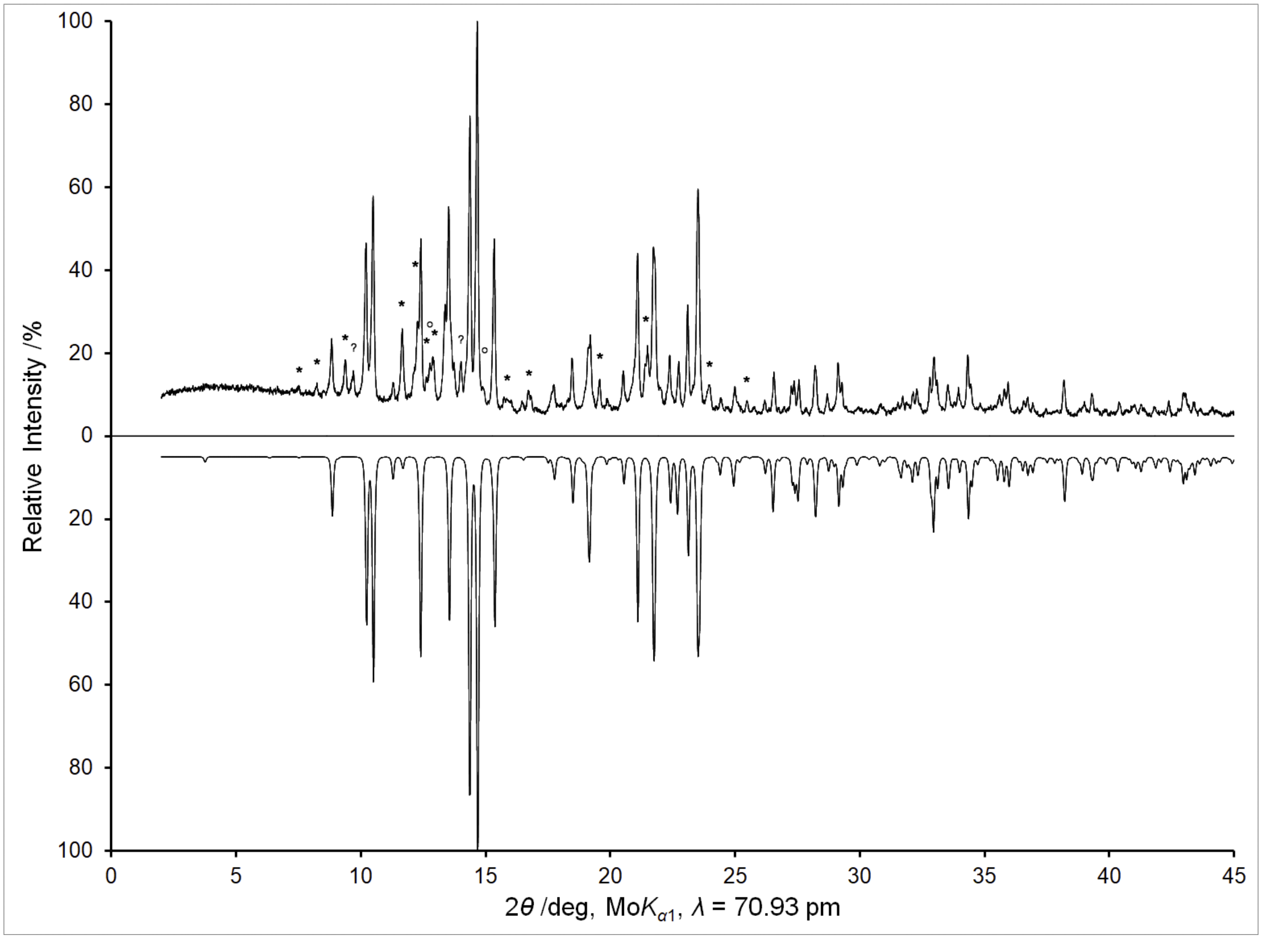

Figure S1: Experimental powder X-ray diffraction pattern of $Pr_5(BO_4)_{3-x}(BO_3)_x(F,OH)_{2.67}O_{0.28}$ ($x \approx 1.6$) (top) in comparison to the theoretical powder pattern simulated from single-crystal X-ray diffraction data (bottom). Reflections marked with asterisks originate from $Pr_{12}B_{11}O_{31}F_7$[3] and reflections marked with circles are caused by a small amount of remaining educt. Residual reflections (the most evident marked with question marks) belong to an unidentified side phase.

Vibrational Spectroscopy

Figure S2 shows the infrared (IR) spectrum of $Pr_5(BO_4)_{3-x}(BO_3)_x(F,OH)_{2.67}O_{0.28}$ ($x \approx 1.6$) in the range of 500 – 4000 cm^{-1}. The spectrum was measured from a bulk sample of the reaction product, containing only HP-PrOF[4] as major side product detectable by powder diffraction measurement, with a Bruker ALPHA-P "Platinum-ATR" FT-IR spectrometer. Background subtraction and atmospheric correction were applied. 240 scans of the sample were acquired with a resolution of 4 cm^{-1}. As HP-PrOF does not show any strong absorption bands above 600 cm^{-1}, interference with the relevant regions of the IR spectrum can be excluded.

The absorption band of $Pr_5(BO_4)_{3-x}(BO_3)_x(F,OH)_{2.67}O_{0.28}$ ($x \approx 1.6$) are typical for borates exhibiting BO_4 tetrahedra and BO_3 groups.[5] The strong bands around 600 and 700 cm^{-1} can be attributed to in-plane and out-of-plane bending vibrations of BO_3 groups, respectively.[5a, 5b, 6] Bands in the range of 800 – 1100 cm^{-1} are to be assigned to stretching vibrations within tetrahedral BO_4 groups,[5a, 7] while absorption bands around 1250 cm^{-1} arise from stretching vibrations of trigonal planar BO_3 groups.[6-7, 8] The FT-IR spectrum clearly confirms the existence of both BO_4 and BO_3 groups in the crystal structure of $Pr_5(BO_4)_{3-x}(BO_3)_x(F,OH)_{2.67}O_{0.28}$ ($x \approx 1.6$).

In the region of 3000 to 3500 cm^{-1}, no significant absorption bands could be detected. While one cannot exclude the existence of any H_2O or OH groups in the crystal structure of $Pr_5(BO_4)_{3-x}(BO_3)_x(F,OH)_{2.67}O_{0.28}$ ($x \approx 1.6$) in this way, it confirms that, if any, only small amounts of H_2O and OH groups are present in the crystal structure.

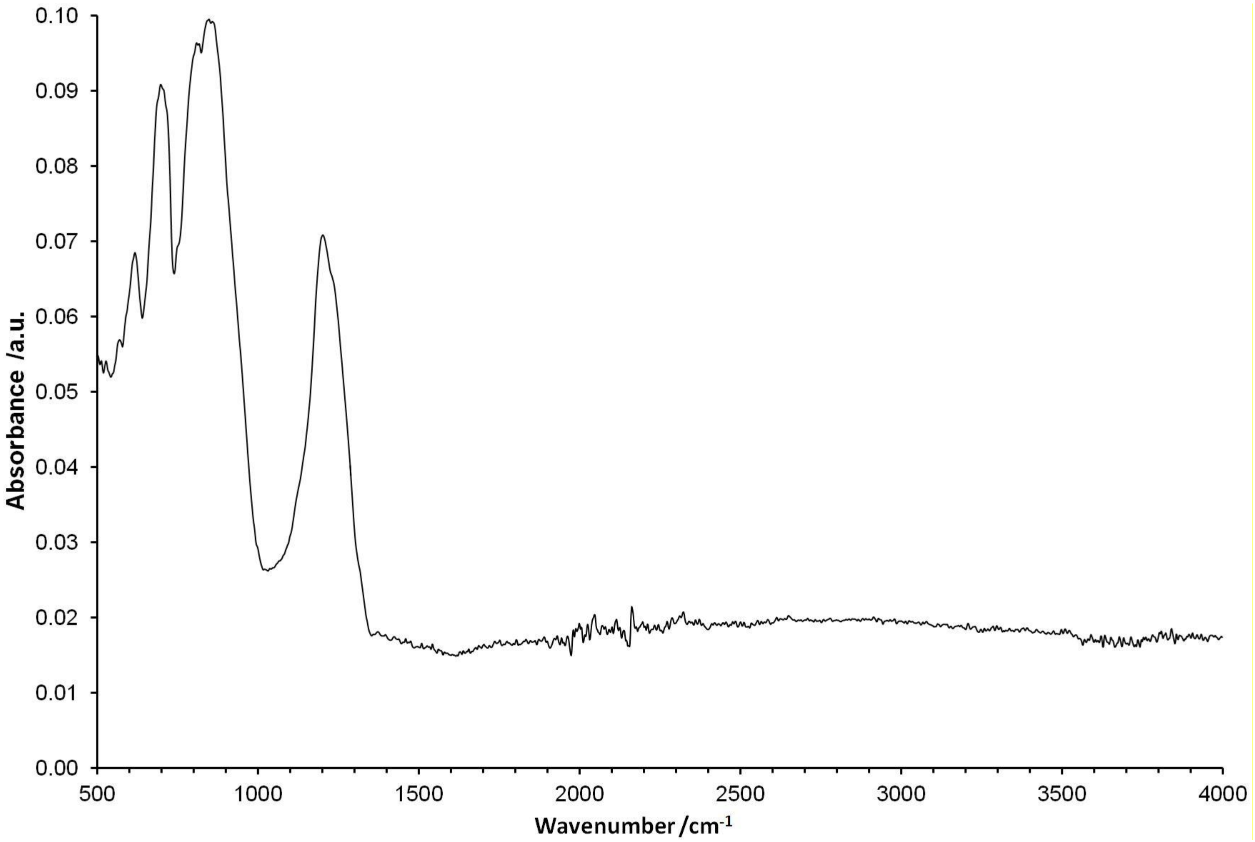

Figure S2: FT-IR spectrum of a bulk sample of $Pr_5(BO_4)_{3-x}(BO_3)_x(F,OH)_{2.67}O_{0.28}$ ($x \approx 1.6$).

References

[1] a) D. Walker, M. A. Carpenter, C. M. Hitch, *Am. Mineral.* **1990**, *75*, 1020-1028; b) D. Walker, *Am. Mineral.* **1991**, *76*, 1092-1100; c) H. Huppertz, *Z. Kristallogr. - Cryst. Mater.* **2004**, *219*, 330-338.

[2] a) G. M. Sheldrick, *SADABS* v. 2014/5, Bruker/Siemens Area Detector Absorption Correction Program, Bruker AXS Inc., Madison, Wisconsin, USA, **2014**; b) Bruker, *SAINT* v. 8.34a, Bruker AXS Inc., Madison, Wisconsin, USA, **2013**.

[3] A. Pitscheider, PhD thesis, Universität Innsbruck (Austria), **2011**.

[4] M. Glätzle, M. Schauperl, C. Hejny, M. Tribus, K. R. Liedl, H. Huppertz, *Z. Anorg. Allg. Chem.* **2016**, *642*, 1134-1142.

[5] a) J. P. Laperches, P. Tarte, *Spectrochim. Acta* **1966**, *22*, 1201-1210; b) G. Heymann, K. Beyer, H. Huppertz, *Z. Naturforsch., B: J. Chem. Sci.* **2004**, *59*, 1200-1208; c) M. Glätzle, H. Huppertz, *Z. Naturforsch., B: J. Chem. Sci.* **2013**, *68*, 635-642.

[6] K. Machida, H. Hata, K. Okuno, G. Adachi, J. Shiokawa, *J. Inorg. Nucl. Chem.* **1979**, *41*, 1425-1430.

[7] a) M. Ren, J. H. Lin, Y. Dong, L. Q. Yang, M. Z. Su, L. P. You, *Chem. Mater.* **1999**, *11*, 1576-1580; b) M. Glätzle, G. Heymann, H. Huppertz, *Z. Kristallogr. - Cryst. Mater.* **2013**, *228*, 449-456; c) M. Glätzle, J. Hoerder Gregor, H. Huppertz, *Z. Naturforsch., B: J. Chem. Sci.* **2016**, *71*, 535-542; d) S. D. Ross, *Spectrochimica Acta Part A: Molecular Spectroscopy* **1972**, *28*, 1555-1561.

[8] W. C. Steele, J. C. Decius, *J. Chem. Phys.* **1956**, *25*, 1184-1188.
